# Клинико-лабораторные особенности макронодулярной двусторонней гиперплазии надпочечников

**DOI:** 10.14341/probl13301

**Published:** 2023-06-30

**Authors:** А. Шевэ, А. Р. Елфимова, Д. Г. Бельцевич

**Affiliations:** Национальный медицинский исследовательский центр эндокринологии; Национальный медицинский исследовательский центр эндокринологии; Национальный медицинский исследовательский центр эндокринологии

**Keywords:** макронодулярная гиперплазия надпочечников, синдром Иценко–Кушинга

## Abstract

**ОБОСНОВАНИЕ:**

ОБОСНОВАНИЕ. Макронодулярная двусторонняя гиперплазия надпочечников (МДГН) — доброкачественное поражение надпочечников, которое в ряде случаев приводит к гиперкортицизму. Ввиду низкой выявляемости, неспецифической, стертой клинической картины и медленного, многолетнего прогрессирования трудно оценить истинную распространенность МДГН. Это объясняет и весьма ограниченные литературные данные. Детальный анализ лабораторных, радиологических показателей, клинической картины, в частности оценка течения коморбидных состояний (артериальной гипертензии (АГ), сахарного диабета (СД), остеопороза), необходим для определения тактики ведения пациентов с МДГН.

**ЦЕЛЬ:**

ЦЕЛЬ. Изучение особенностей клинико-лабораторной картины МДГН у взрослых пациентов, а также поиск факторов, обуславливающих ее клиническую гетерогенность.

**МАТЕРИАЛЫ И МЕТОДЫ:**

МАТЕРИАЛЫ И МЕТОДЫ. Проведено одноцентровое одномоментное исследование. Обследованы 110 пациентов с МДГН, обратившихся в ФГБУ «НМИЦ эндокринологии» в период 2014–2022 гг. Проведен сравнительный и корреляционный анализ гормональных (кортизол в ходе ночного подавляющего теста (ПДТ1), суточный кортизол мочи (СКМ), адренокортикотропный гормон (АКТГ)), биохимических (гликированный гемоглобин), радиологических данных (объем узловой ткани), течения сопутствующих заболеваний (метаболического синдрома, СД, АГ, остеопороза) в трех группах пациентов: с манифестным синдромом Кушинга (СК), с функционально-автономной продукцией кортизола (ФАПК) и коморбидными заболеваниями, МДГН без гормональной активности.

**РЕЗУЛЬТАТЫ:**

РЕЗУЛЬТАТЫ. В исследование включены 110 пациентов, среди них 79,1% женщин, медиана возраста — 60 лет [51; 68]. Доля гормонально-неактивных форм МДГН составила 37,3%, манифестная форма СК выявлена в 25,4% случаев, у остальных пациентов (37,3%) диагностирована ФАПК. По данным гормонального обследования: уровень кортизола в ходе ПДТ1 — 173,8 нмоль/л [86,0; 441,0], АКТГ — 3,35 пг/мл [1,00; 8,00], СКМ — 445,5 нмоль/сут [249,0; 900,0]. Выявлены статистически значимые положительные умеренные корреляции между объемом узловой ткани и уровнем кортизола после ПДТ1 (r=0,40; p<0,001), СКМ (r=0,29; p<0,004), а также отрицательная умеренная корреляция между объемом и уровнем АКТГ (r=-0,40; p<0,001). При анализе распространенности и клинической выраженности коморбидных состояний СД диагностирован у 22 пациентов (53,7%), АГ — у 36 пациентов (87,8%), ожирение и нарушения минерально-костного обмена — 23 (56%) и 3 (7,3%) пациентов. Частота ассоциированных с СК заболеваний (СД, АГ, остеопороз) и значения индекса массы тела статистически значимо не различаются в вышеуказанных группах.

**ЗАКЛЮЧЕНИЕ:**

ЗАКЛЮЧЕНИЕ. По результатам исследования можно сделать вывод, что МДГН является гетерогенной патологией с различными клиническими, гормональными и радиологическими характеристиками. В нашей госпитальной выборке доминируют гормонально-активные формы МДГН. Выявлена корреляция между объемом узловой ткани и степенью гормональной активности МДГН. Полученные результаты подчеркивают трудность в определении четких показаний к хирургическому лечению в группе пациентов с ФАПК.

## Abstract (en)

**BACKGROUND:**

BACKGROUND: Primary bilateral macronodular adrenal hyperplasia (PBMAH) is characterized by bilateral benign adrenocortical lesions, which in some cases lead to Cushing syndrome (CS). Due to the low detection, non-specific, erased clinical picture and slow, long-term progression, it is difficult to assess the true prevalence of PBMAH. This also leads to fairly limited literature data. A detailed analysis of biochemical, imaging parameters, the clinical presentations, in particular, an assessment of the course of comorbidities (arterial hypertension (AH), diabetes (DM), osteoporosis), is necessary to develop an algorithm for managing patients with PBMAH.

**AIM:**

AIM: Analysis of clinical and laboratory characteristics of patients with various forms of PBMAH.

**MATERIALS AND METHODS:**

MATERIALS AND METHODS: A single-center, retrospective, observational, cross-sectional study was carried out. This study included 110 patients with PBMAH who got referred to the National Research Center for Endocrinology in the period from 2013–2023. We carried out comparative and correlation analysis of hormonal (plasma cortisol concentrations after 1 mg dexamethasone (1-mg DST), urinary free cortisol (СКМ), ACTH), biochemical (glycated hemoglobin), radiological data (nodular tissue volume), course of comorbidities (metabolic syndrome, DM, AH, osteoporosis) in three groups of patients: with overt CS, mild autonomous cortisol excess (MACE) and comorbid diseases, and patients with PBMAH without hormonal activity.

**RESULTS:**

RESULTS: Among 110 patients 79.1% were women, median age — 60 [51; 68]. The proportion of hormonally inactive forms of PBMAH was 37.3%, the overt CS and MACE was detected in 25.4 and 37.3% consequently. According to the hormonal ­examination data: the cortisol level during 1-mg DST was 173.8 nmol/l [86.0; 441.0], ACTH — 3.35 pg/ml [1.00; 8.00], СКМ — 445.5 [249.0; 900.0]. Statistically significant positive moderate correlations were found between the volume of nodular tissue and the level of cortisol after PDT1 (r=0.40, p<0.001), СКМ (r=0.29, p<0.004), as well as a negative moderate correlation between the volume and the level of ACTH (r=-0.40, p<0.001). When analyzing the prevalence and clinical severity of comorbid conditions, DM was diagnosed in 22 (53.7%), AH in 36 (87.8%), obesity and osteoporosis — 23 (56%) and 3 (7.3%) patients. There was no statistically significant difference in the prevalence of CS-associated diseases among the above groups (p=0.56).

**CONCLUSION:**

CONCLUSION: PBMAH is a heterogeneous pathology with different clinical, hormonal, and imaging characteristics. A correlation was found between the volume of nodular tissue and the degree of hormonal activity of PBMAH. The obtained results emphasize the difficulty in determining clear indications for surgical treatment in the group of patients with MACE. The radicality of proposed surgical procedure should be weighed against its potential complications.

## ОБОСНОВАНИЕ

В структуре заболеваемости населения России доминируют заболевания, связанные с нарушениями углеводного обмена, а также сердечно-сосудистой системы. Повышение артериального давления (АД) является ключевым фактором развития преждевременной смерти, причиной смерти 10 млн и инвалидизации более 200 млн человек в мире. В большинстве случаев артериальная гипертензия (АГ) сочетается с метаболическими нарушениями. Эти две патологии являются взаимоотягощающими, ускоряющими поражение различных органов-мишеней, что приводит к развитию сердечной, почечной недостаточности, нарушению мозгового кровообращения и, соответственно, к ранней инвалидизации и преждевременной смерти. Распространенность АГ среди населения России продолжает увеличиваться с каждым годом, на данный момент достигнув уровня 47%. У части пациентов данной группы нарушения вызваны патологией эндокринных желез, в частности — надпочечников. Отмечена прямая корреляция между наличием гормонально-активных опухолей надпочечников и ранним дебютом, более тяжелым течением не только АГ, но также сахарного диабета (СД) и метаболических нарушений.

Резкое увеличение применения методов лучевой диагностики, в первую очередь компьютерной томографии (КТ), связанное с эпидемией COVID-19, привело к росту выявляемости опухолей надпочечников. Последующее обследование пациентов привело к более ранней диагностике гормонально-активных форм заболеваний.

Макронодулярная двусторонняя гиперплазия надпочечников (МДГН) — патология надпочечников, которую относят к заболеваниям коркового слоя надпочечников. Она характеризуется образованием двусторонних гиперплазированных, иногда гигантских надпочечников за счет наличия множественных узлов более 1 см, которое в ряде случаев приводит к избыточной продукции кортизола.

МДГН может встречаться как в изолированной форме, так и в рамках генетически детерминированного синдрома.

Синдромальная форма возникает при доминантно наследуемых заболеваниях, которые обусловлены наличием инактивирующей мутации зародышевой линии в различных генах-супрессорах опухолей. Достаточно редко МДГН может встречаться при синдроме множественной эндокринной неоплазии 1 типа (MEN1), семейном аденоматозном полипозе (APC), наследственном лейомиоматозе и почечно-клеточном раке (FH). Синдром МакКьюна–Олбрайта, вызванный постзиготической активирующей мутацией гена GNAS1, кодирующего альфа-субъединицу G-белка, классически диагностируется как триада признаков: фиброзная дисплазия костей, преждевременное половое созревание и пятна на коже цвета «кофе с молоком». Результатом GNAS1-мутации является активация внутриклеточных сигнальных каскадов (аденилатциклазный путь), которая приводит к стимуляции стероидогенеза [1].

При изолированной форме наблюдаются как спорадические, так и генетически детерминированные случаи. Изменение стероидогенеза при МДГН связывают и с некоторыми молекулярными изменениями. У преобладающей части пациентов с МДГН (до 90%) активация пути цАМФ-зависимой протеинкиназы А может быть результатом стимуляции лигандами-агонистами, отличными от рецепторов адренокортикотропного гормона (АКТГ), связанных с G-белком. При МДГН выявляются два типа рецепторов: аберрантные и эутопические. К аберрантным относятся рецепторы вазопрессина 2 и 3 типа, серотонина 7 типа, глюкозозависимого инсулинотропного пептида, α2А-адренорецепторы, в то время как рецепторы вазопрессина 1 типа, лютеинизирующего гормона/хорионического гонадотропина человека, β-адренорецепторы, серотонина 4 типа и лептина в норме обнаруживаются в коре надпочечников, однако при МДГН отмечается их гиперэкспрессия [2]. В ходе диагностических стимуляционных тестов выявлено, что до 86% пациентов имеют множественную рецепторную экспрессию рецепторов, результатом которых является аберрантная реакция кортизола [3–9].

При МДГН наблюдается феномен интраадреналовой продукции АКТГ. Активация аберрантных рецепторов приводит к паракринной секреции АКТГ, тем самым образуется аутокринная/паракринная петля, которая способствует гиперпластическим изменениям в надпочечниках и секреции стероидов [10].

В 2013 г. при полногеномном секвенировании генома Assie и соавт. впервые описали инактивирующую мутацию в гене-супрессоре опухолевого роста ARMC5, расположенном на длинном плече 16 хромосомы (16p11.2) [11]. Идентифицировано более 80 различных мутаций ARMC5, распределенных по всей кодирующей последовательности, однако на сегодняшний день частых мутаций или «горячих» точек не выявлено. По данным различных исследований, у пациентов с МДГН, сопровождающейся манифестным синдромом Кушинга (СК), распространенность мутации ARMC достигает 50%, в то время как у пациентов с субклиническим течением МДГН она выявляется гораздо реже (~11%) [12].

МДГН, как правило, не имеет яркой клинической картины, за исключением форм, сопровождающихся манифестным СК. Ей свойственны различные неспецифические клинические проявления, многолетнее и скрытое, но прогрессирующее течение. Хроническая гиперсекреция кортизола приводит к различным метаболическим нарушениям, среди них ожирение, нарушение углеводного обмена, дислипидемия и АГ. Вялотекущее течение этой патологии приводит к несвоевременной диагностике и, в большинстве случаев, постановке диагноза МДГН уже на этапе наличия осложнений, вызванных многолетним хроническим воздействием избытка кортизола.

## ЦЕЛЬ ИССЛЕДОВАНИЯ

Целью работы является изучение клинических, биохимических и радиологических характеристик МДГН и поиск факторов, влияющих на течение данного заболевания.

## МАТЕРИАЛЫ И МЕТОДЫ

## Место и время проведения исследования

Место проведения. ФГБУ «НМИЦ эндокринологии Минздрава России».

Время исследования. Январь 2014–Декабрь 2022 г.

## Изучаемые популяции (одна или несколько)

Для включения в исследование рассматривались пациенты старше 18 лет мужского и женского пола. Все участники исследования проходили обследование или обращались за медицинской консультацией в ГНЦ ФГБУ «Научный медицинский исследовательский центр эндокринологии» Минздрава РФ в период 2012–2022 гг. Первоначально для включения в исследования были отобраны 165 пациентов с двусторонними множественными образованиями надпочечников.

Критерии включения:

Критерии исключения:

## Способ формирования выборки из изучаемой популяции (или нескольких выборок из нескольких изучаемых популяций)

Сплошной

## Дизайн исследования:

## Методы

Всем пациентам выполнялась КТ органов брюшной полости и забрюшинного пространства на мультидетекторном компьютерном томографе Optima CT660 фирмы General Electric. Толщина срезов составляла 0,625 мм. Границы сканируемой области — от диафрагмы до седалищных бугров. При необходимости выполнялось внутривенное болюсное контрастирование неионным контрастным веществом (КВ) (Йомепрол 400 мг/мл). Оценка артериальной и венозной фаз контрастирования осуществлялась на 5-й и 20-й секундах с момента достижения порогового контрастирования аорты. Оценку отсроченной фазы проводили на 10-й минуте от начала введения КВ.

Измерение каждого узлового образования надпочечника осуществлялось не менее чем в трех сечениях (длина, ширина и высота). Расчет объема опухолевой ткани осуществлялся по формуле для нахождения объема сфер (Vсф):

Vсф= 4/3 πr3,

где π — число π, а r — радиус.

Для узловых образований по типу эллипсоида расчет объема проводился по формуле:

Vэлл= 4/3 πabc,

где a, b, c — это полуоси эллипсоида. Подсчет общего объема узлового поражения надпочечников производился путем сложения объемов всех узлов с двух сторон. При конгломератных образованиях надпочечников оценка объема осуществлялась по максимальным суммарным размерам.

На этапе радиологического обследования проводилась дифференциальная диагностика МДГН с кистами и миелолипомами. Для кистозных образований характерно полное отсутствие накопления КВ. Диагноз двусторонних миелолипом устанавливался при наличии образований с участками жировой ткани, которые имеют резко отрицательную нативную плотность (менее 0 единиц Хаунсфилда (HU)), и миелоидными компонентами, для которых характерна высокая нативная плотность (>20 HU). При наличии отягощенного онкологического анамнеза исключалась возможность вторичного метастатического поражения надпочечников. В первую очередь это осуществлялось на основании данных КТ (высокая нативная плотность, быстрый рост образований), а также повышения специфических онкомаркеров. При вышеуказанных патологиях также отсутствует характерная для МДГН гиперплазия ткани надпочечника вне узла.

Диагностика гормональной активности проводилась в соответствии с отечественными клиническими рекомендациями Минздрава России по дифференциальной диагностике инциденталом надпочечников.

Для подтверждения гормональной активности МДГН проводилось определение уровня кортизола в ходе ночного подавляющего теста с 1 мг дексаметазона (ПДТ1). Методика проведения ПДТ1: прием 1 мг дексаметазона между 23 и 24 ч, на следующее утро забор крови на кортизол в интервале между 8:00–9:00. Исследование уровня кортизола выполнено на электрохемилюминесцентных анализаторах фирмы Roche (Cobas 6000 Modulee601) стандартными наборами фирмы F. Hoffmann-La Roche Ltd). Согласно действующим клиническим рекомендациям, точка сut-off определена на уровне 50 нмоль/л.

При положительном результате ПДТ1 (кортизол >50 нмоль/л) дополнительно всем пациентам исследовался уровень свободного кортизола в суточной моче иммунохемилюминесцентным методом на аппарате Vitros Eci с предварительной экстракцией эфиром. Методика сбора биологического материала: сбор суточной мочи осуществлялся в течение 24 ч в одну емкость. Осуществлялся учет общего объема мочи. Пациенту было рекомендовано соблюдать обычный питьевой режим, отменить прием препаратов, влияющих на содержание кортизола в образце суточной мочи, а также диуретиков, избегать физических и эмоциональных стрессов. Референсные значения лаборатории: свободный кортизол суточной мочи — 60–413 нмоль/сут.

Исследование уровня АКТГ выполнено на электрохемилюминесцентных анализаторах фирмы Roche (Cobas 6000 Modulee601) стандартными наборами фирмы F. Hoffmann-La Roche Ltd. Забор крови из периферической вены выполнялся натощак в промежуток с 8:00 до 09:00. Референсные значения лаборатории: АКТГ — 7,2–66 пг/мл. Дифференциальная диагностика между АКТГ-зависимыми и -независимыми формами СК проводилась по уровню АКТГ. При снижении уровня АКТГ менее 5 пг/мл устанавливался АКТГ-независимый характер гиперкортицизма.

Манифестная форма СК устанавливалась при:

При отсутствии лабораторно подтвержденной гормональной активности и наличии характерных изменений (узловая гиперплазия надпочечников) по данным КТ устанавливалась «гормонально-неактивная форма МДГН»

Исключение двусторонних феохромоцитом проводилось пациентам с высокоплотными образованиями по данным КТ (>20 HU) на основании нормальных значений суточной экскреции метилированных катехоламинов. При наличии АГ проводилась первичная диагностика ПГА — исследование уровня альдостерона, ренина и калия.

При подозрении на ВДКН оценивались уровни АКТГ (при данной патологии и отсутствии компенсации данный показатель превышает 150 пг/мл) и кортизола (менее 500 нмоль/л). В случае получения вышеуказанных результатов дальнейшее обследование проводилось в соответствии с действующими клиническими рекомендациями.

План обследования пациента включал сбор:

Масса тела измерялась с помощью медицинских весов, рост измерялся с точностью до сантиметра с помощью настенного стадиометра. ИМТ рассчитывался по формуле:

ИМТ=m/h2,

где m — масса тела (кг), а h — рост (м).

При осмотре оценивалось наличие/отсутствие типичных клинических проявлений СК: увеличение массы тела, «кушингоидный тип ожирения», багровые стрии, матронизм (яркий румянец на щеках), гиперандрогенизм, мышечная слабость, атрофия мышц конечностей.

Диагноз артериальной АГ устанавливался при многократном повышении АД выше 140/90 мм рт. ст. Всем пациентам с сопутствующей АГ проводилась консультация кардиолога для установления степени гипертонической болезни (ГБ), стадии АГ и инициации/коррекции антигипертензивной терапии.

При наличии у пациента нецелевых показателей глюкозы плазмы крови натощак (>6,1 ммоль/л), для исключения наличия нарушений углеводного обмена проводился пероральный глюкозотолерантный тест. При установлении диагноза СД определялся уровень гликированного гемоглобина (HbA1c). HbA1c определялся методом высокоэффективной жидкостной хроматографии на анализаторе D10 (BioRad, США) с использованием наборов того же производителя по стандартной методике; метод сертифицирован NGSP (National Glycohemoglobin Standardization Program). Референсные значения 4–6%.

Рентгеновская денситометрия проводилась на денситометре Lunar iDXA (GE Healthcare, США). Критерии установления диагноза остеопороза:

## Статистический анализ

Статистический анализ выполнен в пакете прикладных программ Statistica v. 13 (TIBCO Software Inc., США). Описательная статистика количественных переменных представлена медианами, первым и третьим квартилями в виде Me [Q1; Q3], категориальных — абсолютными и относительными частотами в виде n (%). Доверительные интервалы (ДИ) для частот рассчитаны методом Клоппера–Пирсона.

Сравнение двух независимых групп для количественных данных выполнялось с помощью критерия Манна–Уитни (U-тест). Сравнение 3 независимых групп для количественных данных выполнялось с помощью критерия Краскела–Уоллиса (H-тест). Частоты бинарных признаков сравнивались между собой с помощью критерия χ2 и точного критерия Фишера.

Корреляционный анализ количественных параметров выполнялся с помощью метода Спирмена.

Критический уровень статистической значимости при проверке статистических гипотез принят равным 0,05. Поправка Бонферрони применялась путем коррекции критического уровня значимости (Р0) при множественных сравнениях. Статистические тенденции — значения между Р0 и 0,05 соответственно.

## Этическая экспертиза

Локальным этическим комитетом ФГБУ «НМИЦ эндокринологии» Минздрава России вынесено заключение об одобрении проведения клинического исследования, протокол № 17 Заседания локального этического комитета от 28 октября 2020 г. Все пациенты подписывали добровольное информированное согласие на участие в исследовании.

## РЕЗУЛЬТАТЫ

Из 123 пациентов, первоначально обратившихся на прием или проходивших обследование/лечение в ФГБУ «НМИЦ эндокринологии» Минздрава России, в результате первичного скрининга были отобраны 110 больных с МДГН. Характеристика пациентов представлена в таблице 1. На этапе клинического обследования из 123 исключены 10 человек, среди которых выявлены ВДКН (n=3), беременность (n=1), метастатическое поражение надпочечников (n=4), другие гормонально-активные образования надпочечников (миеломы, кисты, лимфомы) (n=5).

**Table:** Таблица 1. Характеристика пациентов с МДГН (n=110)Table 1. Characteristics of patients with MDGN (n=110)

Признак	N	Me [ Q1; Q3] / n (%)
Мужской пол	44	10 (22,7%)
Возраст, лет	44	53 [ 45; 64]
Манифестный СК	44	21 (47,7%)
ИМТ, кг/м2	44	30,8 [ 27,5; 34,4]
Объем опухолевой ткани, см3	44	27,9 [ 17,9; 49,2]
Кортизол после ПДТ1, нмоль/л	44	496,5 [ 295,5; 691,5]
АКТГ, пг/мл	44	1,00 [ 0,00; 2,05]
АКТГ	АКТГ <1 пг/мл	44	23 (52,3%)
АКТГ 1,1–5 пг/мл	21 (47,7%)
UFC, нмоль/сут	43	992,0 [ 474,0; 1895,0]
СД	44	18 (40,9%)
HbA1с, %	22	7,0 [ 6,3; 7,7]
Количество сахароснижающих препаратов	0	44	31 (70,4%)
1	9 (20,4%)
2	2 (4,5%)
3	2 (4,5%)
Инсулины	18	4 (22,2%)
АГ	44	42 (95,4%)
Стадия АГ	1	42	1 (2,3%)
2	39 (92,8%)
3	2 (4,6%)
Степень ГБ	1	42	1 (2,3%)
2	27 (64,2%)
3	14 (33,3%)
Количество антигипертензивных препаратов	42	3 [ 2; 4]

Далее пациенты были разделены на соответствующие три группы в зависимости от гормональной активности. Был проведен сравнительный анализ среди трех групп пациентов: с манифестным СК, с ФАПК и коморбидными состояниями (АГ, СД, ожирение, остеопороз) и с МДГН без гормональной активности (табл. 2).

**Table:** Таблица 2. Сравнительный анализ пациентов с различными формами МДГН (n=110)Table 2. Comparative analysis of patients with various forms of MDGN (n=110)

Признак	Манифестный СК (группа 1)	ФАПК + коморбидные заболевания (группа 2)	МДГН без гормональной активности (группа 3)	р	p,post-hoc
N	Me [ Q1; Q3] / n (%)	N	Me [ Q1; Q3] / n (%)	N	Me [ Q1; Q3] / n (%)
Мужской пол	28	4 (14,3)	41	9 (22,0)	41	10 (24,4)	0,5862	-
Возраст, лет	28	51[ 43; 62]	41	61[ 53; 64]	41	63[ 57; 70]	<0,0011	р1–2=0,015 р1–3<0,001 р2–3=0,311
ИМТ, кг/м2	28	29,9[ 26,7; 33,0]	40	30,8[ 26,9; 35,0]	36	28,0[ 25,1; 33,9]	0,5602	-
Объем узловой ткани, см3	28	27,9[ 14,3; 48,4]	41	24,6[ 13,5; 40,5]	41	13,6[ 5,6; 20,9]	<0,0011	р1–2=1,000 р1–3<0,001 р2–3=0,004
Кортизол в ходе ПДТ1, нмоль/л	28	540,0[ 295,0; 718,0]	41	192,0[ 102,0; 490,0]	41	79,0[ 54,0; 163,0]	<0,0011	р1–2=0,011 р1–3<0,001 р2–3<0,001
АКТГ, пг/мл	28	1,00[ 0,01; 2,26]	41	2,10[ 1,00; 3,30]	41	9,00[ 7,50; 14,00]	<0,0011	р1–2=0,851 р1–3<0,001 р2–3<0,001
СКМ, нмоль/сут	27	816,0[ 484,0; 1607,0]	39	452,0[ 269,0; 900,0]	32	260,5[ 179,6; 413,0]	<0,0011	р1–2=0,044 р1–3<0,001 р2–3=0,007
HbA1с, %	16	6,0[ 5,6; 6,8]	28	7,1[ 5,9; 8,2]	20	6,5[ 5,9; 7,6]	0,0832	-
СД	28	8 (28,6)	41	22 (53,7)	41	15 (36,6)	0,0892	-
Кол-во сахароснижающих препаратов	0	16	9 (56,3)	23	8 (34,8)	19	5 (26,3)	0,4082	-
1	3 (18,8)	9 (39,1)	7 (36,8)
2	3 (18,8)	3 (13,0)	6 (31,6)
3	1 (6,3)	3 (13,0)	1 (5,3)
Инсулины	16	1 (6,3)	25	4 (16,0)	19	2 (10,5)	0,6272	-
АГ	28	27 (96,4)	41	36 (87,8)	41	32 (78,1)	0,0872	-
Стадия АГ	1	27	1 (3,7)	36	0 (0,0)	32	1 (3,1)	0,0193	р1–2=1,000 р1–3=0,062 р2–3=0,064
2	23 (85,2)	30 (83,3)	18 (56,3)
3	3 (11,1)	6 (16,7)	13 (40,6)
Степень АГ	1	27	1 (3,7)	36	3 (8,3)	32	7 (21,9)	0,2492	-
2	19 (70,4)	25 (69,4)	19 (59,4)
3	7 (25,9)	8 (22,2)	6 (18,8)
Количество антигипертензивных препаратов	27	3 [ 2; 3]	35	3 [ 1; 3]	33	2 [ 1; 3]	0,8812	-
Остеопороз	23	8 (35,8)	29	3 (10,3)	30	5 (16,7)	0,0772	-

С учетом поправки Бонферрони Р0=0,003 статистически значимые различия выявлены по возрасту, объему узлового поражения, уровню кортизола в ходе ПДТ1, АКТГ и СКМ, а также выраженность АГ различалась на уровне статистической тенденции. Таким образом, пациенты с гормонально-неактивной формой МДГН были старше и характеризовались меньшим объемом образования, меньшим уровнем кортизола после ПДТ1 и СКМ и большим значением АКТГ. Частота ассоциированных с СК заболеваний (СД, АГ, остеопороз) и значения ИМТ статистически значимо не различаются в вышеуказанных группах.

С целью выявления корреляций между объемом узловой ткани и гормональными показателями был проведен соответствующий анализ (табл. 3).

**Table:** Таблица 3. Корреляционный анализ между объемом опухолевой ткани и гормональными показателями (n=110)Table 3. Correlation analysis between tumor tissue volume and hormonal parameters (n=110) Поправка Бонферрони Р0=0,05/3=0,017.

Признак	N	R	р
Объем, см3, и кортизол после ПДТ1, нмоль/л	110	0,44	<0,001
Объем, см3, и АКТГ, пг/мл	110	-0,40	<0,001
Объем, см3, и СКМ, нмоль/сут	98	0,29	0,004

В результате корреляционного анализа выявлены статистически значимые положительные умеренные корреляции между уровнем кортизола в ходе ПДТ1, СКМ и объемом узлового поражения надпочечников, а также отрицательная умеренная корреляция между объемом и уровнем АКТГ.

Дополнительно проведен сравнительный анализ 4 подгрупп пациентов в зависимости от уровня АКТГ:

Результаты анализа приведены в таблице 4.

**Table:** Таблица 4. Сравнительный анализ пациентов с различными уровнями АКТГ (n=110)Table 4. Comparative analysis of patients with different levels of ACTH (n=110)

Признак	АКТГ <1 пг/мл (группа 1)	АКТГ 1,1–5 пг/мл (группа 2)	АКТГ 5,1–7,2 пг/мл (группа 3)	АКТГ >7,3 пг/мл (группа 4)	р	p,post-hoc
N	Me [ Q1; Q3] / n (%)	N	Me [ Q1; Q3] / n (%)	N	Me [ Q1; Q3] / n (%)	N	Me [ Q1; Q3] / n (%)
ИМТ, кг/м2	30	30,7[ 27,6; 34,7]	36	30,0[ 25,7; 34,5]	8	28,0[ 26,8; 30,1]	30	28,3[ 25,0; 34,0]	0,8091	-
Клинические признаки СК	30	15 (50%)	37	12 (32%)	11	0 (0%)	32	4 (13%)	0,0013	р1–2=1,000 р1–3=0,018 р1–4=0,013 р2–3=0,265 р2–4=0,507 р3–4=1,000
АГ	30	29 (97%)	37	32 (87%)	11	9 (82%)	32	25 (78%)	0,1922	-
СД	30	12 (40%)	37	17 (46%)	11	4 (36%)	32	12 (38%)	0,8862	-
Остеопороз	24	5 (21%)	26	6 (23%)	8	0 (0%)	24	5 (21%)	0,6133	-

С учетом поправки Бонферрони Р0=0,01 статистически значимые различия обнаружены лишь по частоте проявлений клинических признаков гиперкортицизма. В группе пациентов с АКТГ<1 пг/мл клинические проявления СК выявлялись значительно чаще, чем в группе пациентов с АКТГ>7,3 пг/мл. В группе пациентов с АКТГ=5,1–7,2 пг/мл пациенты с клиническим СК отсутствовали.

## ОБСУЖДЕНИЕ

Впервые МДГН была описана группой авторов М.А. Kirschner и соавт. в 1964 г. [13]. На протяжении многих лет предложено множество терминов, описывающих данное заболевание: гигантская макронодулярная гиперплазия надпочечников, АКТГ-независимая макронодулярная гиперплазия надпочечников, первичная макронодулярная неоплазия коры надпочечников и др. [2–4]. На данный момент нет четко принятой дефиниции МДГН. Как правило, под этим термином понимают значительное увеличение надпочечников за счет наличия множественных узелков, размер которых превышает 1 см, сопровождающееся избыточной секрецией кортизола [5][6]. Между тем ряд авторов относят к МДГН гиперпластические процессы, которые приводят к гиперсекреции не только глюкокортикостероидов, но и минералокортикоидов [14]. В нашей работе диагноз МДГН выставлялся в первую очередь на основании данных радиологического обследования. В исследование были включены и гормонально-неактивные двусторонние множественные образования надпочечников, так как прогрессирующий рост узлов в течение жизни в конечном итоге приводит к гормональной автономии и нарастанию клинической симптоматики.

В настоящий момент общепринятая классификация МДГН надпочечников отсутствует. В зависимости от клинического течения заболевания можно выделить:

Cредний возраст исследуемой группы составил 60 лет, с преобладанием пациентов женского пола (соотношение женщин и мужчин ~5:1), что в целом соответствует данным ранее опубликованных исследований [15]. Преобладание гормонально-активных форм МДГН (62,7%) обусловлено госпитальным характером выборки пациентов. В популяционных исследованиях на долю МДГН без гормональной активности приходится 58–73,7% [9][10]. Пациенты с гормонально-активными формами МДГН имеют, как правило, более яркое клиническое тяжелое течение сопутствующих заболеваний, что влечет за собой более высокую обращаемость пациентов к врачу и, как следствие, более тщательное обследование, что обуславливает преобладание в нашем исследовании пациентов с ФАПК и манифестным СК. Более того, принимая во внимание ограниченную репрезентативность выборки, полученные результаты невозможно экстраполировать на общую популяцию.

Мы получили закономерные результаты гормонального обследования: в группе МДГН без гормональной активности получены низкие показатели кортизола в ходе ПДТ1 и СКМ при нормальных показателях АКТГ (р<0,001). Статистически значимых различий между гормональными показателями в группах ФАПК и манифестным СК не выявлено.

В группе пациентов с ФАПК отмечено, что у более половины исследуемых (53,6%) в отсутствие подавления кортизола в ходе ПДТ1 при сниженном АКТГ уровень СКМ находился в пределах референсного интервала. Существует несколько возможных объяснений данного явления. Концепция первой теории основывается на возможном влиянии на концентрацию свободного кортизола различных факторов, находящихся непосредственно в сыворотке крови. Помимо кортизол-связывающего белка, или транскортина (ТК), сыворотка крови содержит такие факторы, как цитокины и сывороточные факторы роста, которые приводят к искусственно измененным результатам гормонального обследования [16]. На сегодняшний день метаболомных исследований у пациентов с МДГН не проводилось. Актуальным представляется исследование роли ТК и других факторов в течении МДГН.

Вторая гипотеза базируется на данных о неэффективном производстве кортизола при МДГН. При гистологическом исследовании макроузлы надпочечников состоят преимущественно из больших клеток (спонгиоцитов). Преобладание таких крупных прозрачных клеток является причиной относительно неэффективного стероидогенеза, однако это нивелируется при значительном увеличении клеточной массы в гиперплазированной коре надпочечников, что в итоге приводит к гиперсекреции глюкокортикостероидов [17]. По результатам исследования Н.Р. Hsiao и соавт. пациенты с МДГН имеют повышенную экскрецию 17-гидроксикортикостероидов (17OHS), имеющих гидроксильную группу в положении 17 стероидной структуры, в которую входят кортизол, кортизон, 6-гидроксикортизол, тетрагидрокортизол, аллотетрагидрокортизол и тетрагидрокортизон. Таким образом, у пациентов с МДГН экскретируются с мочой различные метаболиты глюкокортикоидов, которые не обнаруживаются при исследовании СКМ. Необходимо проведение дополнительных исследований диагностической ценности 17OHS для валидизации данного метода [8]. Проведение мультистероидного анализа крови и мочи у пациентов МДГН перспективно с точки зрения выявления новых, более специфичных диагностических тестов для данной патологии.

В ходе корреляционного анализа выявлены статистически значимые положительные умеренные корреляции между объемом узловой ткани и уровнем кортизола в ходе ПДТ1 (r=0,40; p<0,001), СКМ (r=0,29; p<0,004), а также отрицательная умеренная корреляция между объемом узловой ткани и уровнем АКТГ (r=-0,40; p<0,001). В многочисленных исследованиях зарубежных авторов было продемонстрировано, что при проведении сцинтиграфии с NP-59 более высокое накопление радиофармпрепарата происходит в доминирующем по размеру надпочечнике [14–20]. Прямая зависимость объема узловой ткани с гормональными показателями свидетельствует о том, что увеличение объема узловой ткани при МДГН приводит к увеличению стероидогенеза, супрессии АКТГ и, как следствие, ухудшению течения СК. Этот факт обуславливает нарастание клинической симптоматики при динамическом наблюдении пациентов с МДГН.

Основным методом лечения гормонально-активных форм МДГН на данный момент является оперативное вмешательство, наиболее распространенное — односторонняя адреналэктомия доминирующего по размеру надпочечника [18].

Особую клиническую трудность в определении показаний к хирургическому лечению представляют пациенты с ФАПК и сопутствующими коморбидными заболеваниями. Эта группа пациентов имеет лабораторно подтвержденный эндогенный гиперкортицизм, при этом сниженный, но не супрессированный уровень АКТГ (1–5 пг/мл). На сегодняшний день нет данных о степени влияния супрафизиологического уровня кортизола на течение коморбидных заболеваний, а также является ли избыток кортизола первопричиной развития данных состояний. При принятии решения об оперативном лечении не рекомендуется основываться только на показателях лабораторного обследования, в особенности при несупрессированном уровне АКТГ. У части больных зафиксировано колебание уровня АКТГ в течение периода наблюдения, что свидетельствует о частично сохранной регуляции стероидогенеза гипофизом.

В нашем исследовании был проведен сравнительный анализ среди различных групп пациентов в зависимости от уровня АКТГ (группа 1 — АКТГ менее 1 пг/мл, группа 2 — 1,1–5 пг/мл, группа 3 — 5–7,2 пг/мл, группа 4 — более 7,3 пг/мл). Статистически значимые показатели получены по частоте клинических явлений СК. Так, в группе с АКТГ=5,1–7,2 пг/мл ни у одного пациента не было выявлено признаков гиперкортицизма.

В качестве показаний к оперативному лечению нами рассматривается тяжесть течения коморбидных заболеваний (АГ, СД, ожирение, минерально-костные нарушения), в частности наличие/отсутствие их компенсации, а также объем лекарственной терапии. При анализе распространенности и клинической выраженности сопутствующей патологии у пациентов с ФАПК СД диагностирован в 53,7% (22/41), АГ — в 87,8% (36/41) случаев. В свою очередь, ожирение и нарушения минерально-костного обмена выявлены у 23/41 (56%) и 3/41 (7,3%) пациентов соответственно, что в целом согласуется с более ранними литературными данными [19]. Не выявлено статистически значимых различий в распространенности ассоциированных с СК заболеваний (СД, АГ, остеопороз) и ИМТ у групп с гормонально-активными и неактивными формами МДГН. Полученные результаты еще раз подчеркивают трудность в формировании четких показаний к хирургическому лечению в группе пациентов с ФАПК. При удовлетворительной компенсации АГ и СД целесообразна выжидательная тактика с 6-месячным динамическим контролем гормональных показателей, а также гликемии, АД и массы тела. При прогрессирующем снижении уровня АКТГ, ухудшении течения коморбидных состояний рекомендуется оперативное лечение.

## ОГРАНИЧЕНИЯ ИССЛЕДОВАНИЯ

Ограничением настоящего исследования является малая выборка пациентов. Пропуски данных обусловлены ретроспективным характером исследования.

## ЗАКЛЮЧЕНИЕ

В настоящем исследовании продемонстрирована МДГН как гетерогенная патология с различными клиническими, гормональными и радиологическими характеристиками. В нашей госпитальной выборке доминируют гормонально-активные формы МДГН. Выявлена корреляция между объемом узловой ткани и степенью гормональной активности МДГН. Полученные результаты подчеркивают трудность в определении четких показаний к хирургическому лечению в группе пациентов с ФАПК. Остаются нерешенными проблемы должной диагностики и разработки персонализированного ведения больных. Требуется более детальное изучение структуры опухолей надпочечников, факторов, влияющих на течение заболевания, с целью более четкого определения тактики ведения, подбора необходимой индивидуальной терапии и ее коррекции.

## ДОПОЛНИТЕЛЬНАЯ ИНФОРМАЦИЯ

Источники финансирования. Работа выполнена в рамках государственного задания «Разработка новых технологий диагностики и мониторинга опухолей коры надпочечников с использованием метаболомных и протеомных технологий». Регистрационный номер 123021300098-7.

Конфликт интересов. Конфликт интересов отсутствует.

Участие авторов. Шевэ А. — вклад автора по критерию 1 — существенный вклад в концепцию и дизайн исследования, анализ и интерпретацию результатов, по критерию 2 — написание статьи; Елфимова А.Р. — вклад автора по критерию 1 — существенный вклад в анализ и интерпретацию результатов, по критерию 2 — внесение в рукопись существенных (важных правок) с целью повышения научной ценности статьи; Бельцевич Д.Г. — вклад автора по критерию 1 — существенный вклад в концепцию и дизайн исследования, анализ и интерпретацию результатов, по критерию 2 — внесение в рукопись существенных (важных правок) с целью повышения научной ценности статьи. Все авторы одобрили финальную версию статьи перед публикацией, выразили согласие нести ответственность за все аспекты работы, подразумевающую надлежащее изучение и решение вопросов, связанных с точностью или добросовестностью любой части работы.
